# Observations on Such Diseases of the Teeth, as Induce Facial Neuralgia, or Tic Douloureux

**Published:** 1850-01

**Authors:** S. P. Hullihen

**Affiliations:** Wheeling


### OBSERVATIONS ON SUCH DISEASES OF THE TEETH, AS INDUCE FACIAL NEURALGIA, OR TIC DOULOUREUX.

BY DR. S. P. HULLIHEN.

That facial neuralgia sometimes occurs from debility, malaria, and such like causes of a constitutional nature, cannot be denied. But that this disease more frequently occurs from some local exciting cause, either about the jaws or teeth, I think can be most clearly demonstrated; and when well understood, often enables the practitioner to search out and to remove the cause of the disease at once, and thereby to effect a cure that he might otherwise vainly endeavor to effect for days and weeks, by any course of general medication.

In treating of this subject I do not hope to remove the cloud of mystery that for ages has hung over the true nature of this disease; neither do I pretend to have discovered causes that have never before been described. But I simply hope to be able to point out such diseases of the teeth as are most prone to induce facial neuralgia, and then to describe the symptoms and appearances which attend such diseases.

Facial neuralgia sometimes arises from the exposure of the nerve or pulp within the internal cavity of a tooth, sometimes from the absorption of poisons, such as arsenic, when placed within the carious cavity of a tooth for the purpose of effecting the destruction of the nerve—sometimes from the absorption of acrid matter, which may be engendered in a carious cavity of a tooth—sometimes from the pressure of a plug upon the nerve of a tooth—sometimes from the formation of matter within the internal cavity of a tooth—sometimes from exostosis, either upon the apex of a root of a tooth, or within its internal cavity.

It also may arise from other local exciting causes, situated in certain parts of the face and head, such as an accumulation of matter in the antrum maxillare—from polypi or other growths within this cavity—from tumors of different kinds elsewhere about the jaws—or upon the internal surface of the skull—and even from diseases in the substance of the brain itself. But such causes are found to exist but rarely, when compared with those that arise from the teeth. However, the disease arising from any other cause than those of the teeth, cannot be properly considered in the limits proposed in this paper.

Although facial neuralgia may originate from any one of the molars or bicuspids, in either jaw, and occasionally even from the cuspidati and incisors, if the patient be greatly predisposed to neuralgic affections; yet the disease originates with the dens sapientiae or wisdom tooth of the lower jaw, at least seventy-five per cent. oftener than with any other tooth in the mouth. The second molar of the under jaw is the tooth next most frequently implicated—then the first molar, and so on. All the teeth of the lower jaw are more prone to produce neuralgia than those of the upper, and strange as it may appear, the biscuspides of all teeth in the upper jaw are found most frequently to produce the disease. The next tooth of the upper jaw most frequently implicated is the first molar—then the second molar—but the third, or wisdom tooth of this jaw is rarely, if ever, the seat of this complaint. Thus in searching after the cause in any case of facial neuralgia, the wisdom tooth of the lower jaw should be first thoroughly examined—then the second molar—then the first—and then the bicuspides of both jaws—then the first molar of the upper jaw, "and so on back to the place of beginning." Should no cause of the complaint be found in the teeth, then the other locations before mentioned should be carefully examined, or inquired into; and lastly, the true condition of the patient's general health.

To detect the several diseased conditions of the teeth that may induce facial neuralgia, requires some experience, and much close observation. Yet in the great majority of cases, each diseased condition will be found to have its own peculiar characteristics sufficiently well marked to make its discovery quite certain. Though some of the conditions may not be found upon the first, second, or even the third examination, (indeed, they may never be discovered unless the examination be properly conducted, and with the most untiring patience,) yet each and every careful exploration will most likely lead more and more near to the true seat and nature of the disease, until both become fully revealed.

The several diseases of the teeth that may give rise to facial neuralgia, the various symptoms, and the pathological condition of each disease, I will now endeavor to describe.

I have before remarked that diseased teeth in the under jaw were more likely to produce facial neuralgia than those in the upper. This of course depends upon anatomical relations. Then again, that with the wisdom teeth of the lower jaw, the disease originated more frequently than with all the rest of the teeth in the mouth. This depends partly on the great proneness of this class of teeth to decay, but more particularly upon the proximate relations of these teeth to the great branch of the inferior maxillary nerve, and the Gasserion ganglion. The cause of the disease originating with the upper bicuspides more frequently than with any other teeth of the upper jaw, cannot be so satisfactorily explained; perhaps it may be attributed to the more frequent decay of this class of teeth than of any other save that of the first molars, if indeed they are even entitled to the exception.

The exposure of the dental nerve is a very common cause of facial neuralgia, and the disease, when left to run its course, generally subsides upon the suppuration of the nerve. At first the pain is greatly aggravated by any foreign or irritating substance getting into the carious cavity, at the same time the seat of the painful sensation may be more or less remote from the tooth or nerve so irritated. Then again the pain may be located for a few moments in the affected tooth—then it may dart away into the ear, temple, and scalp—or first into one tooth and then into another—and then into all the teeth of both jaws on the affected side—and then in an instant to other associated parts. But there is always uneasiness enough in the proper tooth to indicate the seat and cause of the complaint.

If such a tooth be extracted and examined, the nerve may be found in any intermediate state, from a slight inflammation, up to the suppurative process, depending much upon the violence and duration of the complaint. In such cases, upon the extraction of the tooth, the neuralgia disappears.

The most fearful cases of facial neuralgia sometimes arise from the absorption of arsenic; an article which of late years, has been much employed for the purpose of destroying the nerves of painful teeth. In all cases where the dental nerve is freely exposed, arsenic may be used without danger of producing neuralgia; but in cases where there is only tenderness in the bone of the tooth, or where there exists but a small fissure, leading from the bottom of the carious cavity to that of the nerve, if arsenic be applied, in a few hours more or less of it will be absorbed, the nerve becomes inflamed, and a most painful case of neuralgia will be the result.

Yet in such cases, the tooth may be no more involved in the paroxysms of pain than any other tooth on that side of the head. But if the tooth be extracted, and the nerve examined, it will be generally found in a high state of inflammation, a kind of inflammation, too, that I have never known to run into suppuration.

After such a tooth is extracted, the neuralgic pains will frequently continue for days and weeks before they finally subside.

Facial neuralgia not unfrequently takes place as though it originated from the absorption of acrid or poisonous matter, engendered in the carious cavity of a tooth. In such cases the disease may exist for a long time without the affected tooth being suspected as the cause—however, when the patients attention has been once directed to the teeth, they will generally discover that the paroxysms of pain occur more frequently, and tarry longer in the affected tooth than in any other on that side of the head. I say any other tooth, for it should not be forgotten that in facial neuralgia, let it originate from whatever cause it may, all the teeth on the affected side are liable at times to be more or less involved in the pain, and this painful sensation may be continually changing from one tooth to another, to the sound as well as the diseased. This condition of things exposes the patients very often to have the wrong tooth extracted, and whenever a mistake of this kind is once made, both patient and practitioner are very apt to conclude that all the pains in the teeth are deceptive, and that the true cause of suffering springs from some other source.

If a tooth affected in the manner just described, be extracted and examined, the nerve will be found involved in a low grade of inflammation, chronic of course, and one that is not likely to run very speedily into suppuration.

Whenever a tooth affected in this way is extracted, the neuralgia either immediately subsides, or begins to decline, and will finally disappear in a few days.

Facial neuralgia very frequently takes place from the confinement of matter within the internal cavity of a tooth. The nerve of a tooth very often suppurates from the irritation it receives from the near approach of caries, from fractures, or from the pressure of a plug, and like causes, when in fact the nerve itself is in no way laid bare. The matter in such cases being prevented from escaping through an opening in the crown of the tooth, as it always does when any such opening exists, soon begins to ooze out through the foramen in the apex of a root, and neuralgia to a greater or less extent is the consequence.

The first symptoms attending a tooth about to be affected in this way, is pain, whenever hot or cold fluids pass over it—soon the tooth begins to be sore to the touch, and appears a little loose, and longer than the rest. About this period, some slight neuralgic pains usually commence to dart along the course of the nerves to the temple, ear, and the side of the head, and to the teeth of both jaws. The pains gradually become more and more severe, until an abscess forms, which is the most usual way for this affection of the teeth to terminate. From the very first, the soreness of the tooth will always indicate the seat and true cause of the complaint.

If a tooth effected in this way be extracted and examined at an early stage of the complaint, the nerve will be found suppurating at the point most exposed to irritation. If later, the entire nerve may be destroyed, and still later, a small sac may be forming, or already formed upon the apex of the fang most implicated.

In all such cases the neuralgia subsides immediately upon the extraction of the teeth.

The very worst forms of facial neuralgia very frequently arise from dental exostoses. This deposit of bony matter may attack the roots of teeth either externally or internally. If externally, it forms upon the apex of the root. If internally, it forms in the nerve canal of a fang, either in teeth where the entire crown is worn away, or when the same kind of action is induced at the neck of the tooth, from the absorption of the gum and alveolar process. The external formation upon the apex of the root, or the internal at the neck of the tooth, are the only deposites, so far as I know, that ever give rise to facial neuralgia. The deposit that takes place upon the wearing away of the crown of a tooth, often prevents, but never, I believe, causes pain. Dental exostoses sometimes attack the roots of teeth which, to all appearancce, are perfectly sound; it however much more frequently forms upon the roots of teeth that are diseased.

The existence of this disease is often extremely difficult to detect. Whenever it is located upon the apex of a root, a pretty sharp blow upon the tooth with a small but heavy instrument, will most generally bring on a paroxysm of pain, and thus discover the seat of the disease. But to effect this, it is necessary that the blow should be made in such a way as to make the growth produce an increased, as well as a sudden irritation. Thus if the growth is deposited around the point of the fang, the blow must be given on the face, or masticating surface of the tooth. If upon one side of the root, the blow must be given upon the side of the crown, and on the same side that the growth may be located on the fang. If the growth be situated within the internal cavity of a tooth, then it is determined with still greater difficulty. But if the gum and alveolar process have been absorbed to a considerable extent from about a tooth, and that tooth situated where it is likely to produce neuralgia, and if very cold, or very hot water taken into the mouth provokes paroxysms of pain, such indications are always sufficient to warrant the extraction of the tooth.

If roots of teeth affected with exostosis externally, be examined, the nerve will sometimes be found in a healthy state, at other times perfectly destroyed. The growth may vary from the least perceptible enlargement, to that of a small rifle ball. If internally, the canal will be more or less filled up a little above the neck of the tooth, and the nerve will in such cases, have the appearance of being too small for the remainder of the canal.

Facial neuralgia arising from exostosis, quickly subsides upon the removal of the cause, and I need scarcely add, that this can only be done by the successful removal of the affected root.

But it may be asked, have you not confounded facial neuralgia with common tooth-ache. I answer, in all cases where the greatest amount of suffering is confined to one tooth alone, I call that tooth-ache, without any reference to the cause. But where a tooth is but slightly implicated in pain, where the pain is greatest in the face, temple, or scalp, and where it occurs in paroxysms, or is periodical, I call that facial neuralgia, let it arise from whatever cause it may. The same cause, *in kind* that may produce only tooth-ache in one individual, in another may occasion all the horrors of facial neuralgia.

WHEELING, Jan. 10th, 1850.

